# Reactive Oxygen Species and Mitochondrial DNA Damage and Repair in BCR-ABL1 Cells Resistant to Imatinib

**DOI:** 10.1089/biores.2015.0022

**Published:** 2015-07-01

**Authors:** Janusz Blasiak, Grazyna Hoser, Jolanta Bialkowska-Warzecha, Elzbieta Pawlowska, Tomasz Skorski

**Affiliations:** ^1^ Department of Molecular Genetics, University of Lodz, Lodz, Poland.; ^2^ Department of Clinical Cytobiology, Medical Center for Postgraduate Education, Warsaw, Poland.; ^3^ Department of Infectious and Liver Diseases, Medical University of Lodz, Lodz, Poland.; ^4^ Department of Orthodontics, Medical University of Lodz, Lodz, Poland.; ^5^ Department of Microbiology and Immunology, School of Medicine, Temple University, Philadelphia, Pennsylvania.

**Keywords:** BCR-ABL1, DNA repair, imatinib resistance, mitochondrial DNA damage/repair, reactive oxygen species

## Abstract

Imatinib revolutionized the therapy of chronic myeloid leukemia (CML), but the resistance to it became an emerging problem. We reported previously that CML cells expressing the *BCR/ABL1* fusion gene, accumulated a high level of reactive oxygen species (ROS) due to deregulated mitochondrial electron transport chain, which in turn led to genomic instability, resulting in imatinib resistance. In the present work, we hypothesize that imatinib-resistant cells may show higher instability of mitochondrial DNA (mtDNA) than their sensitive counterparts. To verify this hypothesis, we checked the ROS level and mtDNA damage and repair in model CML cells sensitive and resistant to imatinib and exposed to doxorubicin (DOX), a DNA-damaging agent. The extent of endogenous ROS in imatinib-resistant cells was higher than in their sensitive counterparts and DOX potentiated this relationship. ROS level in cells with primary resistance, which resulted from the T315I mutation in *BCR/ABL1*, was higher than in cells with acquired resistance. DOX-induced mtDNA damage in T315I imatinib-resistant cells was more pronounced than in imatinib-sensitive cells. All kinds of cells were repairing mtDNA damage with similar kinetics. In conclusion, imatinib-resistant cells can show increased instability of mtDNA, which can result from increased ROS production.

## Introduction

The *BCR-ABL1* oncogene results from the t(9;22)(q34;q11) reciprocal translocation leading to a fusion of a part of the *BCR* gene on chromosome 9 with the *ABL1* gene on chromosome 22.^1^ The product of *BCR-ABL1* expression, the BCR-ABL1 protein, has enhanced tyrosine kinase activity, activates several signaling pathways, and confers growth and proliferation advantages, which are typical for cancer cells.^2^

BCR-ABL1 is a hallmark of chronic myeloid leukemia (CML), which therapy has been revolutionized by imatinib, a tyrosine kinase inhibitor (TKI), which has changed CML from a fatal disorder into a chronic disease.^1^ Imatinib binds the adenosine triphosphate (ATP)-binding pocket in BCR/ABL1, inhibiting its kinase activity by blocking its binding by ATP, its cofactor. However, resistance to imatinib has become an emerging problem in CML therapy, which is only partly resolved by second and third generations of TKIs.^3^

Several mechanisms may contribute to imatinib resistance in CML cells, including changes in *BCR-ABL1* expression, mutation(s) in the *BCR-ABL1* gene preventing imatinib binding, deregulation of imatinib cellular export/import, and switching on BCR-ABL1-independent prosurvival mechanisms.^4^ Many mutations in the *BCR-ABL1* gene have been identified to disrupt imatinib binding by changing essential BCR-ABL1 residues involved in contact with imatinib or preventing BCR-ABL1 from adopting an optimal conformation for imatinib binding.

A substitution of threonine by isoleucine at amino acid position 315 (the T315I mutation) is of a special significance because it is most frequently found in imatinib-resistant CML patients.^5^ Moreover, the same amino acid substitution, but at other positions, was found also in the c-Kit and PDGFRα kinases in imatinib-resistant patients with gastrointestinal stromal tumors and hypereosinophilic syndromes, respectively.^2^ The 315 tyrosine may be replaced with phenylalanine with similar effect. Although the involvement of this mutation in imatinib resistance is well established, the question about its significance for CML malignant progression remains open.^3^

In general, imatinib-resistant cells display resistance against imatinib-induced apoptosis.^6^ The intrinsic apoptotic pathway includes mitochondrial outer membrane permeabilization as a major step in apoptosis stimulated by DNA damage or endoplasmic reticulum stress. Imatinib-resistant CML cells are characterized by several mitochondrial dysfunctions, which may be targeted to overcome the resistance.^7^ Therefore, mitochondria may be involved in imatinib-induced apoptosis in CML cells and imatinib resistance.

Mitochondrial mutagenesis may be tightly associated with stability of the organelle and with mitochondria-related apoptosis signaling. Mitochondrial DNA (mtDNA) is considered more prone to mutagenesis than its nuclear counterpart (nDNA). This follows from the proximity of the mitochondrial respiratory chain (MRC), which is the site of oxidative phosphorylation and produces reactive oxygen species (ROS) as its normal by-product. We previously showed that BCR-ABL1 induced ROS that might contribute to imatinib resistance.^8^ ROS may damage mitochondrial genes of MRC, resulting in accelerated ROS production and accumulation of damage to mtDNA.

Although mitochondria display multiple mtDNA repair pathways, including base excision repair (BER), mismatch repair, single-strand break repair, and some mechanisms of recombinational repair, the efficacy of these systems is low compared to the nucleus.^9^

mtDNA containing damage at an extent exceeding the DNA repair capacity of the organelle is degraded in a controlled process. In addition, lack of solid evidence on mitochondrial nucleotide excision repair, the most versatile DNA repair system operating in the nucleus, supports the idea of higher susceptibility of mtDNA to damaging factors than nDNA. Therefore, apoptosis signaling, which is crucial for imatinib therapeutic action, through mitochondria may be more prone to DNA damage and repair than corresponding nuclear signaling.

This hypothesis, along with mitochondrial dysfunction reported in imatinib-resistant leukemic cells, led us to the present study on the role of mitochondrial mutagenesis and ROS production in the resistance of BCR/ABL1-positive cells to imatinib. Another rationale for this study is a high rate of glucose metabolism in imatinib-resistant cells compared with imatinib-sensitive cells, in which a compensatory mechanism in mitochondria may be activated, leading to ROS overproduction.^10–13^

In the present work, we evaluated oxidative stress and assayed the endogenous and induced mtDNA damage, the efficacy of mtDNA repair, and mtDNA copy number in mouse CML model cells sensitive and resistant to imatinib. Imatinib resistance was either primary, resulting from the T315I mutation in the *BCR-ABL1* gene, or secondary (acquired) induced by culturing sensitive cells in increasing concentrations of imatinib. To induce DNA damage, we used doxorubicin (DOX), an anthracycline antibiotic, widely used in anticancer therapy. It targets DNA topoisomerases, inducing DNA double-strand breaks, but it can also produce ROS, which can further damage DNA.^14,15^

Our study is an introduction to the field of the role of mtDNA mutagenesis in cancer transformation induced by BCR-ABL1, in particular in imatinib resistance in CML.

## Materials and Methods

### Chemicals

Imatinib was a kind gift of Novartis (Basel, Switzerland). IMDM medium was purchased from Gibco BRL (Basel, Switzerland). Cell counting kit-8 (CCK-8), DOX hydrochloride, and 2′,7′-dichlorodihydrofluorescein diacetate (DCFH-DA) were obtained from Sigma Chemicals (St. Louis, MO).

### Cells and treatment

The murine 32D clone 3 cell line was transfected with p210 *BCR-ABL1* as described previously.^16^ The cells were transfected with a native BCR-ABL1 or its T315I-mutated variant resistant to imatinib. A part of cells with nonmutated *BCR-ABL1* were cultivated in growing imatinib concentrations at dose escalation of 0.1 μM/week to acquire resistance to 1.0 μM imatinib. Therefore, we used the parental 32D cell line (P) and three 32D BCR-ABL1-transfected cells: sensitive to imatinib (S), a primarily imatinib-resistant line (T315I), and a line with acquired imatinib resistance (AR). The cells were grown in IMDM medium, a modified Dulbecco's medium supplemented with 2 mM l-glutamine, 100 U/mL penicillin, 100 μg/mL streptomycin, and 10% fetal bovine serum, and maintained at 37°C in 5% CO_2_ atmosphere at 100% humidity.

To assess sensitivity/resistance to imatinib, the cells were incubated at 37°C with 1.0 μM imatinib for 24 h. To induce mtDNA damage, the cells were incubated at 37°C with 0.1 μM DOX for 6 h. To observe the kinetics of mtDNA repair, the cells were incubated with 0.2 μM DOX for 6 h at 37°C, then the cells were washed twice in drug-free fresh medium and the extent of DNA damage was estimated immediately and 1, 4, 12, and 24 h thereafter.

### Cell viability

Cell viability was measured colorimetrically with a tetrazolium salt using the CCK-8. Briefly, cells were seeded in 96-well plates at an initial density of 5000/well. After 24 h, the cells were washed with phosphate-buffered saline and the medium was replaced with the appropriate medium containing IM at 1.0 μM. Next, the medium was replaced with a fresh medium containing 10 μL CCK-8 solution and cells were incubated for 1 h. Cell viability was evaluated on the basis of absorbance read with a PowerWave XS microplate reader (Bio-Tek Instruments, Winooski, VT) at 450 nm. Cell viability after DOX treatment was evaluated by the trypan blue exclusion assay.

### Oxidative stress level

The intensity of oxidative stress expressed by the level of ROS production was determined on the basis of oxidative conversion of nonfluorescent DCFH-DA to fluorescent 2′,7′-dichlorofluorescein (DCF).^17^ The cells were seeded into 96-well black plates at a density of 5000/well, and then incubated with DOX as described above. The supernatant was then discarded and replaced with 10 μM DCFH-DA, and cells were cultured in the dark for 45 min. The fluorescence intensity was measured in a Thermo Scientific plate reader, model Fluoroscan Ascent FL (Thermo Scientific, Waltham, MA). Each measurement was repeated five times.

### mtDNA damage and repair

Damage to mtDNA was measured by employing quantitative PCR (QPCR) with a thermostable DNA polymerase blocked by DNA lesions, resulting in a decrease in the amplification of the target sequence in mtDNA.^18^ However, the amount of amplified mtDNA may be related to the template copy number, which corresponds to mtDNA copy number, or is associated with its quality, especially if it is very long. To overcome these obstacles, additional amplification of a small region of mtDNA is performed. This small region is unlikely to be damaged and can serve as polymerase chain reaction (PCR) internal quality control and to normalize the amount of PCR product to relative copy number. Moreover, unlike in classical QPCR with a very long fragment, we deployed semilong run real-time PCR (SL-rtPCR) with a longer amplicon size, about 1000 bp.^19^

This method allows to determine the DNA damage rate in a small exactly defined region of the mitochondrial genome and is free of problems associated with the use of long DNA fragments. To amplify a short fragment, a primer pair of the sequence (all primers sequence in 5′-3′ direction), AACCCACGATCAACTGAAGC (forward) and GTACGATGGCCAGGAGGATA (reverse), positioned within the mitochondrial *ND2* gene and giving a fragment of 82 bp length (chrM: 4058+4139) was used. For the semilong fragment, we used primers of the sequence, (5′-3′) ATCCTCACCTCAGCCAACAA (forward) and TGAGGACTGGAATGCTGGTT (reverse), producing a 1054-bp fragment containing most of the *ND5* gene (chrM 12129–13183).

A fragment of the mouse beta-2 microglobulin gene of 82 bp and sequence, AGAATGGGAAGCCGAACATA (forward) and CCGTTCTTCAGCATTTGGAT (reverse), was amplified as a reference gene. All primers were designated with Primer 3 software (http://simgene.com/Primer3) and synthesized by Sigma Chemicals.

Total, that is, nuclear and mitochondrial, DNA from the cells was isolated with the QIAamp DNa mini Kit (Qiagen, Hilden, Germany) and its purity and concentration were determined spectrophotometrically.

The SL-rtPCR amplifications were run in a thermal cycler model CFX96™ Real-Time PCR Detection System (Bio-Rad Laboratories, Hercules, CA). The amplification of the product was monitored with SYBR Green dye intercalating double-stranded DNA, and the product specificity was determined by melting curve analysis and agarose gel electrophoresis.

Each reaction was conducted in a total volume of 10 μL containing 1× Maxima SYBR Green QPCR Master Mix (Thermo Scientific, West Palm Beach, FL), 500 nM of each primer, and 100 ng of DNA template. SL-rtPCR amplification conditions were determined experimentally and included a preinitial denaturation step at 95°C for 10 min, followed by 40 cycles of 15 sec at 95°C/30 sec at 62°C/75 sec at 72°C for the long product, whereas the short products amplification consisted of a preincubation phase at 95°C for 10 min and 40 cycles of 15 sec at 95°C/30 sec at 59°C/30 sec at 72°C. Each sample was analyzed in triplicate and C_t_ values were obtained directly from the thermocycler software, version 3.0.

Repair of mtDNA was determined on the basis of changes in the extent of mtDNA damage in time.

### mtDNA copy number

Relative copy number of mtDNA was measured by QPCR measuring the ratio of number of copies of a mitochondrial gene to a single-copy nuclear gene.^20^ The mouse mitochondrial gene encoding NADH dehydrogenase subunit 2 (ND2) and nuclear gene encoding beta-2 microglobulin (B2M) were chosen.

The amount of amplification products was obtained from threshold cycle numbers (C_t_) for samples and standard curve that was obtained by measurement of serial dilutions of reference DNA. Reference DNA was DNA extracted from the S cells.

Reactions were performed with the same primers for the 82-bp fragment of the *ND2* gene as in SL-rtPCR, and an 82-bp fragment of the *B2M* gene was amplified with the pair of primers of the sequence, AGAATGGGAAGCCGAACATA (forward) and CCGTTCTTCAGCATTTGGAT (reverse), with the same amplification profile as for the short fragment in SL-rtPCR. The number of copies of mitochondrial and nuclear genes was determined with the thermocycler software based on C_t_ values of standard. Each reaction included 5 μL of the master mix, 1 μL of each primer, 1 μL of DNA matrix, and 2 μL of water.

Each experiment was performed in triplicate and each sample was measured in duplicate. Copy number data generated by thermocycler software were processed for calculation of mtDNA copy number per nuclear genome in each sample.

### Data analysis

DNA damage in DOX-exposed samples was referenced to nonexposed ones. Relative mtDNA damage (*RMD*), expressed as number of mtDNA lesions per 10 kb DNA, was measured by calculating the difference ΔC_t_ for long and short fragments for the 2^*−ΔΔCt*^ method (the comparative *C_t_* method) in correlation with the size of the long fragment after amplification according to the following equation:

\begin{align*}RMD = ( 1 - 2^{- ( \Delta_l - \Delta_s)}) / LFS \end{align*}

where Δ_l_ and Δ_s_ are ΔC_t_ for long and short fragments, respectively, *LFS* is the size of the long fragment in bp.^20,21^

### Statistical analysis

All values in this study are expressed as mean±SEM. The Shapiro–Wilk test was used to assess whether the samples came from a normally distributed population. If this was the case, the differences between means were evaluated by the Student's *t*-test, otherwise the Mann–Whitney test was used. For paired groups, we used either paired *t*-test (normal distribution) or the Wilcoxon signed-rank test (non-normal distribution). The data were analyzed using the STATISTICA (StatSoft, Tulsa, OK) statistical package.

## Results

### Imatinib differentially affects cell viability

**FIG. 1.**
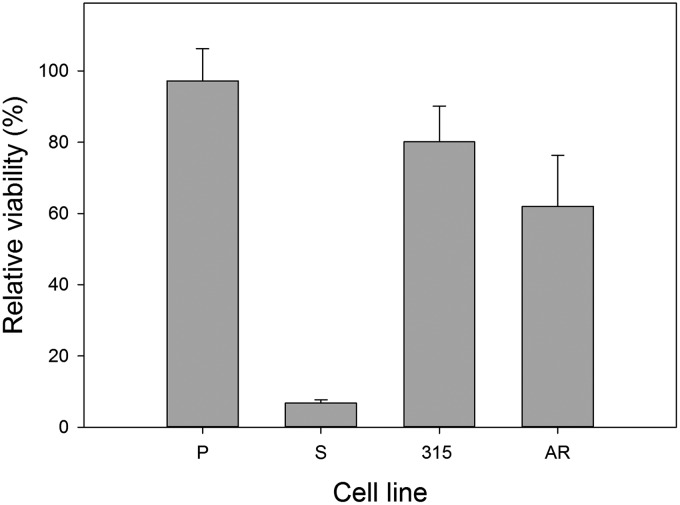
Relative viability of mouse 32D lines: parental (P) and transfected with the *BCR-ABL1* gene: sensitive (S) and resistant to imatinib. The resistance was caused either by T315I mutation in the *BCR-ABL1* gene (315) or by incubation with increasing concentration of imatinib (AR). The cells were incubated with 1 μM imatinib for 24 h at 37°C. The viability was determined spectrophotometrically with cell counting kit-8. Each measurement was repeated in triplicate; error bars denote SEM.

A 24-h incubation with imatinib resulted in almost complete eradication of imatinib-sensitive cells (Fig. 1). The viability of the 315 and AR cells was about 80% and 62%, respectively, and we considered these lines as imatinib resistant.

### Imatinib-resistant cells accumulate more ROS than their sensitive counterparts and DOX potentiates this relationship

**FIG. 2.**
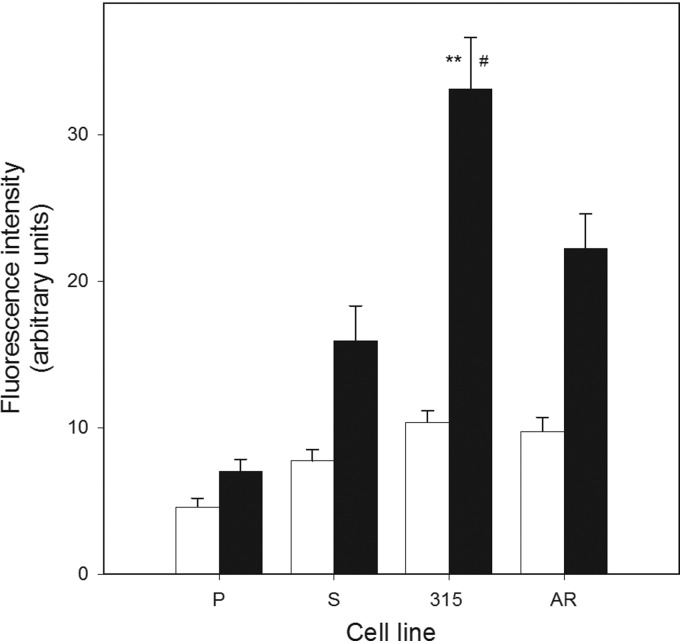
Reactive oxygen species (ROS) levels in mouse 32D cell lines: parental (P) and transfected with the *BCR-ABL1* fusion gene: sensitive (S) and resistant to imatinib in the presence (black bars) or absence (white bars) of 0.1 μM DOX. The resistance resulted either from the T315I mutation in *BCR-ABL1* (315) or from incubation with increasing concentrations of imatinib (AR). ROS level is expressed by the fluorescence of 2′,7′-dichlorofluorescein oxidatively converted from dichlorodihydrofluorescein diacetate. Error bars denote SEM, *n*=5, for each measurement; **p<0.01 compared with S cells, ^#^*p*<0.05 compared with AR cells.

The extent of intracellular ROS in all *BCR-ABL1*-transfected cells, both sensitive and resistant to IM, was significantly higher when compared with 32D parental cells (*p*<0.05) (Fig. 2). The ROS level in 315 cells was considerably higher than in S cells. DOX enhanced ROS in both *BCR-ABL1*-transfected and nontransfected cells (Fig. 2). The presence of BCR-ABL1 further increased the ROS level, and the imatinib-resistant T315I cells displayed significantly higher ROS level than sensitive cells (*p*<0.01) and AR cells (borderline significance). This lack of a substantial difference between the T315I and AR cells suggests that imatinib resistance in AR may result, at least in part, from different mechanism(s) than the T315I mutation.

### Imatinib-resistant T315I cells accumulate more DOX-induced mtDNA damage than their imatinib-sensitive counterparts

**FIG. 3.**
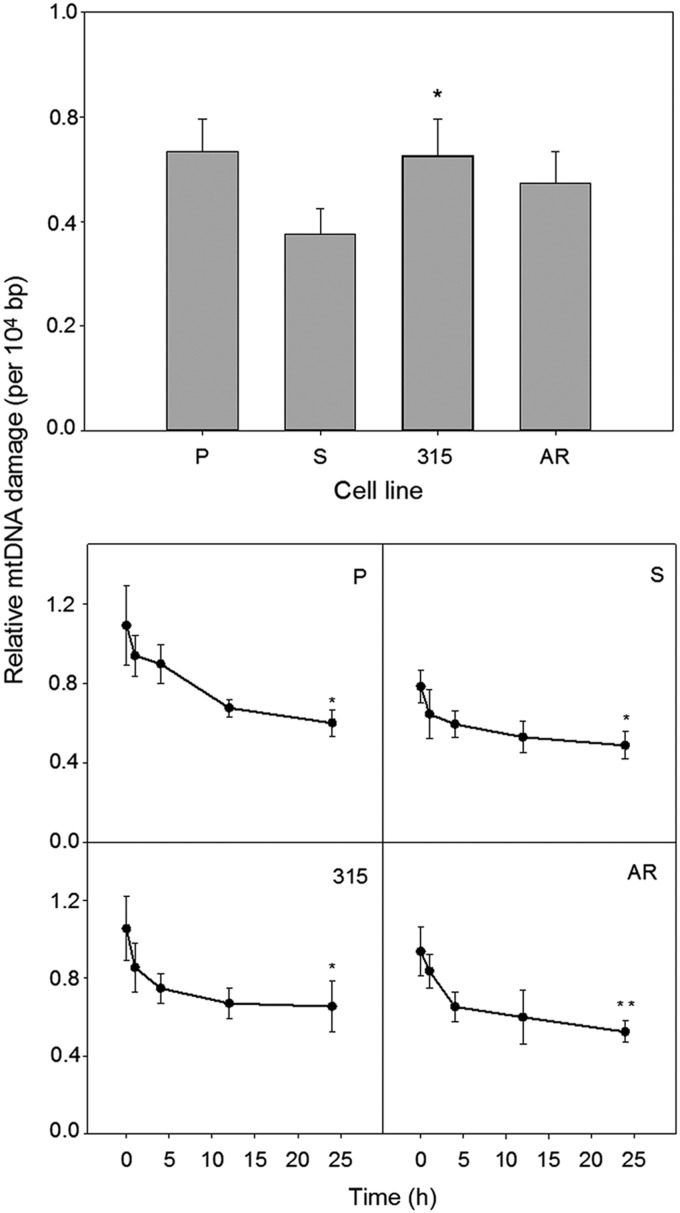
DOX-induced mitochondrial DNA (mtDNA) damage and repair in mouse 32D cell lines: parental (P) and transfected with the *BCR-ABL1* gene: sensitive (S) and resistant to imatinib (315, AR). The resistance resulted either from the T315I mutation in the *BCR-ABL1* gene (315) or from incubation with increasing concentration of imatinib (AR). Cells were incubated for 6 h at 37°C with DOX at 0.1 μM, and damage to mtDNA was quantified by semilong real-time polymerase chain reaction (PCR) (upper panel). In mtDNA repair experiments (lower panel), cells were incubated for 6 h at 37°C with DOX at 0.2 μM, washed, and mtDNA damage was quantified immediately (time 0) and 1, 4, 12, or 24 h thereafter. Error bars denote SEM, *n*=5, for each measurement; upper panel: *p<0.05 compared with S line; lower panel: *p<0.05, **p<0.01 compared with initial (time zero) mtDNA damage.

A 6-h incubation with 0.1 μM DOX induced mtDNA damage in all kinds of 32D cells (Fig. 3). The extent of this damage was roughly comparable with that observed for a positive control, which was 200 μM hydrogen peroxide applied for 30 min (data not shown). DOX-induced mtDNA damage in T315I imatinib-resistant cells was higher than in their imatinib-sensitive, S, counterparts (*p*<0.05). The extent of the damage in AR cells was higher than in S cells, but this difference was not statistically significant. DOX-induced mtDNA damage in S cells was significantly lower (*p*<0.05) than in parental cells.

### Imatinib-resistant and -sensitive cells display similar kinetics of repairing of DOX-induced mtDNA damage

We observed a decrease in time in the extent of mtDNA damage induced by DOX for all cell lines (Fig. 3). It suggests that this damage might have been repaired in all kinds of cells. However, due to a relatively small difference between the extent of mean mtDNA damage at time 0 and 24 h and considerable errors in some time points, no definite conclusion on the DNA repair should be drawn, although the decrease in the extent of mtDNA damage between end and initial points of repair incubation was statistically significant (*p*<0.05 for P, S, and 315 lines, *p*<0.01 for AR line), but its biological relevance is uncertain.

### DOX does not affect mtDNA copy number in imatinib-resistant and -sensitive cells

**FIG. 4.**
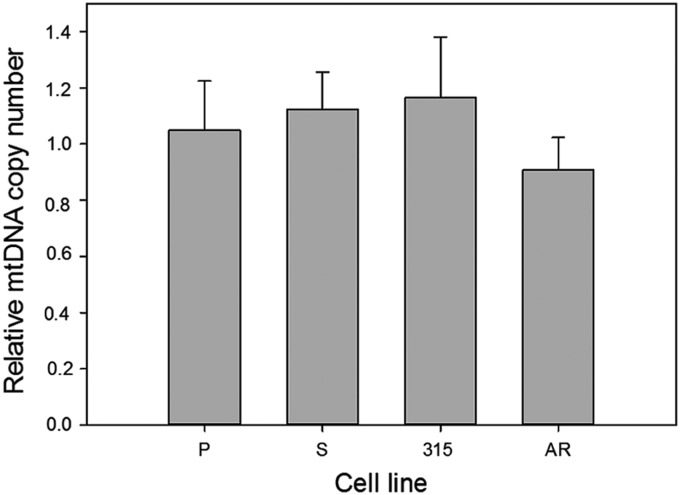
Relative mtDNA copy number in mouse 32D cell lines: parental (P) and transfected with the *BCR-ABL1* gene: sensitive (S) and resistant to imatinib (315, AR). The resistance resulted either from the T315I mutation in the *BCR-ABL1* gene (315) or from incubation of the sensitive cells with increasing concentrations of imatinib (AR). mtDNA copy number was calculated by quantitative real-time PCR of 82-bp fragments of the mitochondrial NADH dehydrogenase subunit 2 (*ND2*) gene and the nuclear beta-2 microglobulin (*B2M*) gene. Presented is the ratio of the copy number in cells exposed for 6 h at 37°C to 0.1 μM DOX and unexposed cells. Error bars denote SEM, *n*=5, for each measurement.

The mean mtDNA copy number in cells exposed to DOX relative to nonexposed cells is presented in Figure 4. Exposure of the cells to 0.1 μM DOX did not result in difference in mtDNA copy number between imatinib-resistant and -sensitive cells. Therefore, one can conclude that these two kinds of cells did not differ in the extent of programmed degradation of mtDNA resulting from DOX-induced damage.

## Discussion

In this work, we focused on the reaction of mtDNA to DNA damage in imatinib-sensitive and -resistant *BCR-ABL1*-expressing cells. We also studied this reaction in the parental cells without the *BCR-ABL1* gene, but they were included only for reference as we did not attempt to study general aspects of the influence of BCR-ABL1 on mitochondrial mutagenesis, although little is known on this subject. As we mentioned in the Introduction section, resistance to imatinib is related to resistance to apoptosis, which can follow one of two basic pathways, extrinsic and intrinsic (mitochondrial), and mtDNA damage may influence mitochondrial signaling, so it may affect apoptosis induced by imatinib or even trigger this process, independently of imatinib.^22,23^

There are several mechanisms of imatinib resistance, including the drug export by α1-acid glycoprotein and its import by hOCT1 protein, as well as activation of signaling pathways leading to BCR-ABL1-independent growth, including Ras/Raf/Mek, PI3K, Stat, and Erk pathways.^4^ We do not exactly know which mechanism underlines the resistance of AR cells and we did not even try to attempt to determine this mechanism as this would require an additional extensive study. Moreover, we believe that the acquired resistance to imatinib may be underlined by various mechanisms, even if it is induced in apparently similar conditions. This is supported by some clinical observations.^3^

BCR-ABL1 stimulates ROS production, enhances oxidative nDNA damage, modulates nDNA repair mechanisms, and increases the rate of mutagenic events in the nucleus.^24^ mtDNA is likely to be more prone to oxidative stress than its nuclear counterpart.^22^

In concordance with previous reports, all cell lines expressing BCR-ABL1 displayed a higher intracellular ROS level than parental cells.^8^ It has been hypothesized that BCR-ABL1-expressing cells may have a leakage in mitochondrial electron transport chain, resulting in ROS overproduction.^25^

We observed a further increase in the ROS level in imatinib-resistant cells compared with imatinib-sensitive cells. There are several limitations and artifacts associated with the DCF assay we employed.^26^ However, we used it just for detection of the level of general oxidative stress associated with increased ROS production and not for measuring the level of any particular ROS. The assay is widely used for this purpose. DOX produced ROS in both *BCR-ABL1*-transfected and nontransfected cells, but the presence of BCR-ABL1 increased the ROS level and the imatinib-resistant 315 cells displayed significantly higher ROS level than sensitive cells.

It is really intriguing that the mutated BCR-ABL1 induced even more ROS than its nonmutated form upon DOX treatment. We can speculate that if normal BCR/ABL1 increases genome instability, its mutated form can further increase it, which may be associated with the decreased ability of the cell to scavenge ROS. This might be somehow similar to the mitochondrial vicious cycle.^27^

Our study on mtDNA repair after DOX treatment suggests that all kinds of cells might try to recover their DNA during a 24-h repair incubation, which led to a decrease in DNA damage (Fig. 4). In this series of experiments, we used a higher concentration of DOX (0.2 μM) than in the study of mtDNA damage (0.1 μM). We tried to use the same drug concentration throughout our entire study, but the effect of mtDNA repair after cell treatment with 0.1 μM DOX was hardly seen (results not shown), so we decided to increase twice the concentration. Therefore, we started from the same concentration of the drug in each cell line and we just showed that the level of DNA damage varied with cell type.

This raises the question whether the same concentration of the drug or rather the same extent of DNA damage should be assumed at the beginning of DNA repair kinetics study. We chose the same DOX concentration as it was difficult to assess the drug concentration to induce the same DNA damage in all cell lines in one experiment. DOX has a broad spectrum of DNA damage it can induce.^28^ However, DOX, possibly thanks to its structure, also stimulates ROS production, which may induce a broad spectrum of DNA damage, first of all oxidative base modifications, especially at moderate DOX concentrations.^29^ Therefore, DNA double-strand breaks (DSBs) repair and BER may be primarily involved in recovering of DNA in cells treated with DOX.

However, the above data were obtained for nDNA and cannot be exactly extrapolated onto mtDNA due to differences in cellular reaction to DNA damage between nuclear and mtDNA, resulting in different susceptibility of mtDNA than nDNA to DOX. DOX, which is a cationic compound, may easily penetrate mitochondria, where it may bind to cardiolipin and disturb the functioning of the respiratory chain, increasing ROS production.^14,15^ DOX may directly target mtDNA to intercalate and induce deletions.^30,31^

Although several facts, some of them mentioned above, suggest that mtDNA should accumulate more oxidative damages than its nuclear counterpart, this issue has not been definitely resolved.^22^ However, there is no doubt that mtDNA is susceptible to oxidative modifications induced by DOX. Therefore, we conclude that mtDNA damage induced by DOX that we observed included DSBs and oxidative modifications to DNA bases and they could have been repaired.

Although our knowledge on DNA repair in mitochondria is far from complete, efficient BER operates in mitochondria and DSBs might be repaired by both nonhomologous end joining and homologous recombination repair as the presence of functional DNA ligase III, Ku80-like, RAD51, and RAD52 proteins was observed in mitochondria.^32^ We can therefore interpret the results obtained after repair incubation (Fig. 3) as representing a biphasic repair process with BER activity, dominating during the first 1–2 hours of repair incubation and DSB repair during the rest of the incubation time.

Searching for the correlation between the ROS level and mtDNA damage in dependence on BCR-ABL1 expression and imatinib resistance is a rather complex issue. First of all, there are substantial differences between each of the two cell lines we used, and especially parental cells are different from the remaining, BCR-ABL1-expressing cells. The results presented for parental cells are only for reference and rather not for an exact comparative analysis.

However, it is known and we showed several times that BCR-ABL1 might stimulate DNA repair, but this repair may be unfaithful.^8^ In other words, BCR-ABL1-stimulated DNA repair may lead to restoring DNA integrity, but it may induce premutagenic changes, mainly alterations in DNA sequence, which were not detected in the assay we applied. Therefore, the results of the PCR-based study indicated the higher number of intact templates for the reaction in the sensitive cells, but these templates could contain an unknown number of changes in DNA sequence and other alterations escaping PCR assay. Hence, despite the lower level of mtDNA damage in BCR-ABL1-expressing imatinib-sensitive cells than in parental cells, these cells may display a higher degree of genomic instability.

However, this DNA repair-stimulating action is apparently different in imatinib-resistant cells and results from an unknown mechanism. However, first of all, comparing the results presented in Figures 2 and 3, one should take into account that cellular ROS production (Fig. 2) may result from the instability of both nuclear and mitochondrial genomes, whereas the extent of DNA damage presented in Figure 3 is for mtDNA only.

The expression and activity of BCR-ABL1 were checked during cell culturing (data not shown), but we did not attempt to establish a relationship between BCR-ABL1 expression/activity, DOX concentration, imatinib resistance, and mtDNA mutagenesis as it would create too many variables for a reliable interpretation.

Contrary to its nuclear counterpart, mtDNA can be degraded when heavily damaged and replaced with a new one, which implies that its copy number may change during genotoxic treatment.^33^ However, it is not a canonical mechanism of mitochondria to cope with excessive or unrepaired damage, but rather as a general mtDNA-specific mechanism to maintain its integrity.

We observed a slight difference in mtDNA copy number between 32D parental and BCR/ABL-expressing cell lines and DOX changed the copy number in various ways in all cell lines. However, the change was not significant and we do not want to interpret it at this stage of our research. There are a few reports on the effect of DOX on the mtDNA copy number, but, similarly to ROS action, no definite conclusion can be drawn.^34,35^ Apparently, a more detailed study of mtDNA copy number in BCR/ABL-expressing and nonexpressing as well as IM-sensitive and -resistant cells is needed.

Our study was performed on the single-cell model, which is a limitation of our research. However, this model is rather unique as it allows studying both primary and secondary imatinib resistance, as well as the reaction of BCR/ABL1-expressing and nonexpressing cells.

## Conclusions

BCR-ABL1-expressing cells resistant to imatinib can show increased instability of mtDNA than their imatinib-sensitive counterparts. This difference may follow from the increased production of ROS in imatinib-resistant cells.

## Abbreviations Used

CML: chronic myeloid leukemia
TKI: tyrosine kinase inhibitor
ATP: adenosine triphosphate
mtDNA: mitochondrial DNA
MRC: mitochondrial respiratory chain
ROS: reactive oxygen species
BER: base excision repair
DOX: doxorubicin
AR: acquired imatinib resistance
PMD: Relative mtDNA damage
DSB: double-strand breaks
